# Supplementary material for: Cryo-EM structures of Banna virus in multiple states reveal stepwise detachment of viral spikes
Source: Nat Commun. 2024 Mar 13;15:2284. doi: 10.1038/s41467-024-46624-x (PMC10937716; doi:10.1038/s41467-024-46624-x)
Supplement: Supplementary file 1 — Supplementary Information [file 41467_2024_46624_MOESM1_ESM.pdf]

Supplementary Information for

Cryo-EM structures of Banna virus in multiple states reveal stepwise detachment of viral spikes

Zhiqiang Li[1,2], Han Xia[1], Guibo Rao[1], Yan Fu[1], Tingting Chong[1,2], Kexing Tian[1,2], Zhiming Yuan[1]*, Sheng Cao[1]*

1 CAS Key Laboratory of Special Pathogens, Wuhan Institute of Virology, Center for Biosafety Mega-Science, Chinese Academy of Sciences, Wuhan 430071, P. R. China

2 University of Chinese Academy of Sciences, Beijing 100049, P. R. China

Zhiqiang Li, Han Xia, and Guibo Rao contributed equally to this work.

*To whom correspondence should be addressed:

Prof. Dr. Sheng Cao, Tel./Fax: +86-27-87198286, Email: caosheng@wh.iov.cn

Prof. Dr. Zhiming Yuan, Tel./Fax: +86-27-87198195, Email: yzm@wh.iov.cn

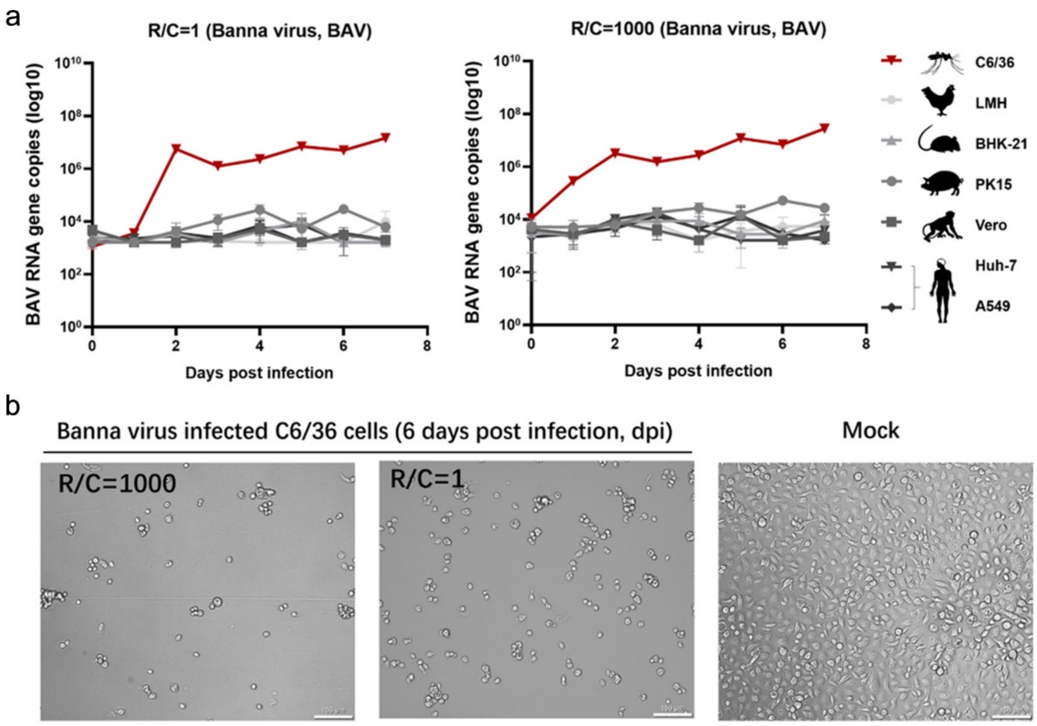

Supplementary Fig. 1 Growth characteristics of Banna virus strain YN15-126-01. The figure was created with BioRender.com.

a, Growth curves of BAV strain YN15-283-01 with different R/C (1 or 1000) in cells derived from mosquito (C6/36), chicken (LMH), and mammals (mouse, pig, monkey, and human).

b, Typical cytopathic effect (CPE), characterized by shrinkage and death, observed in C6/36 cells 6 days post-BAV infection. Scale bar: 100 μm.

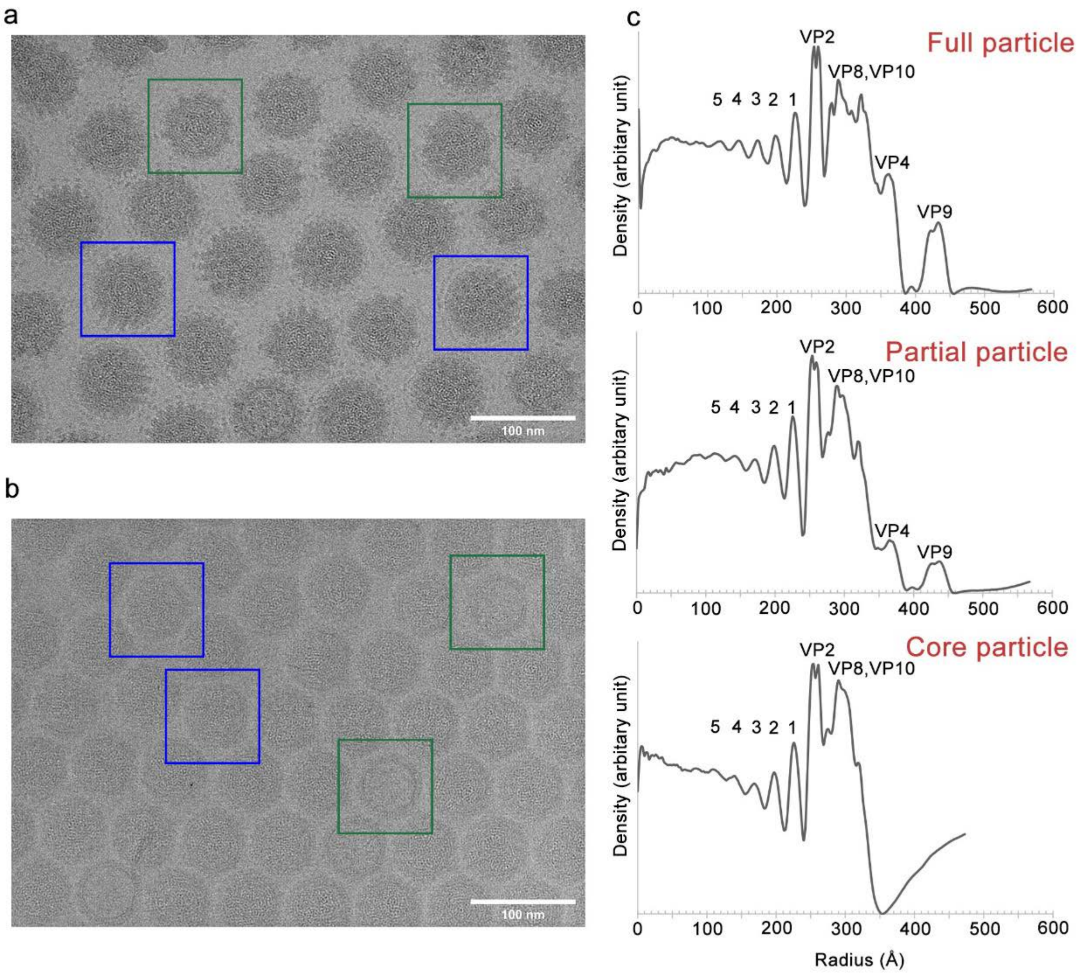

Supplementary Fig. 2 Cryo-EM images of BAV.

a, Cryo-EM images of purified BAV particles. Two representative full particles are indicated by blue boxes, while partial particles are indicated by green boxes.

b, Cryo-EM images of BAV core particles. Two representative core particles are indicated by blue boxes, while empty particles are indicated by green boxes.

c, Virus radial density profiles derived from the cryo-EM reconstructions for full, partial, and core particles. Density peaks for protein shells are labeled by corresponding proteins. dsRNA shells are denoted by numbers, and at least five dsRNA shells are distinguishable for each of the three types of BAV particle.

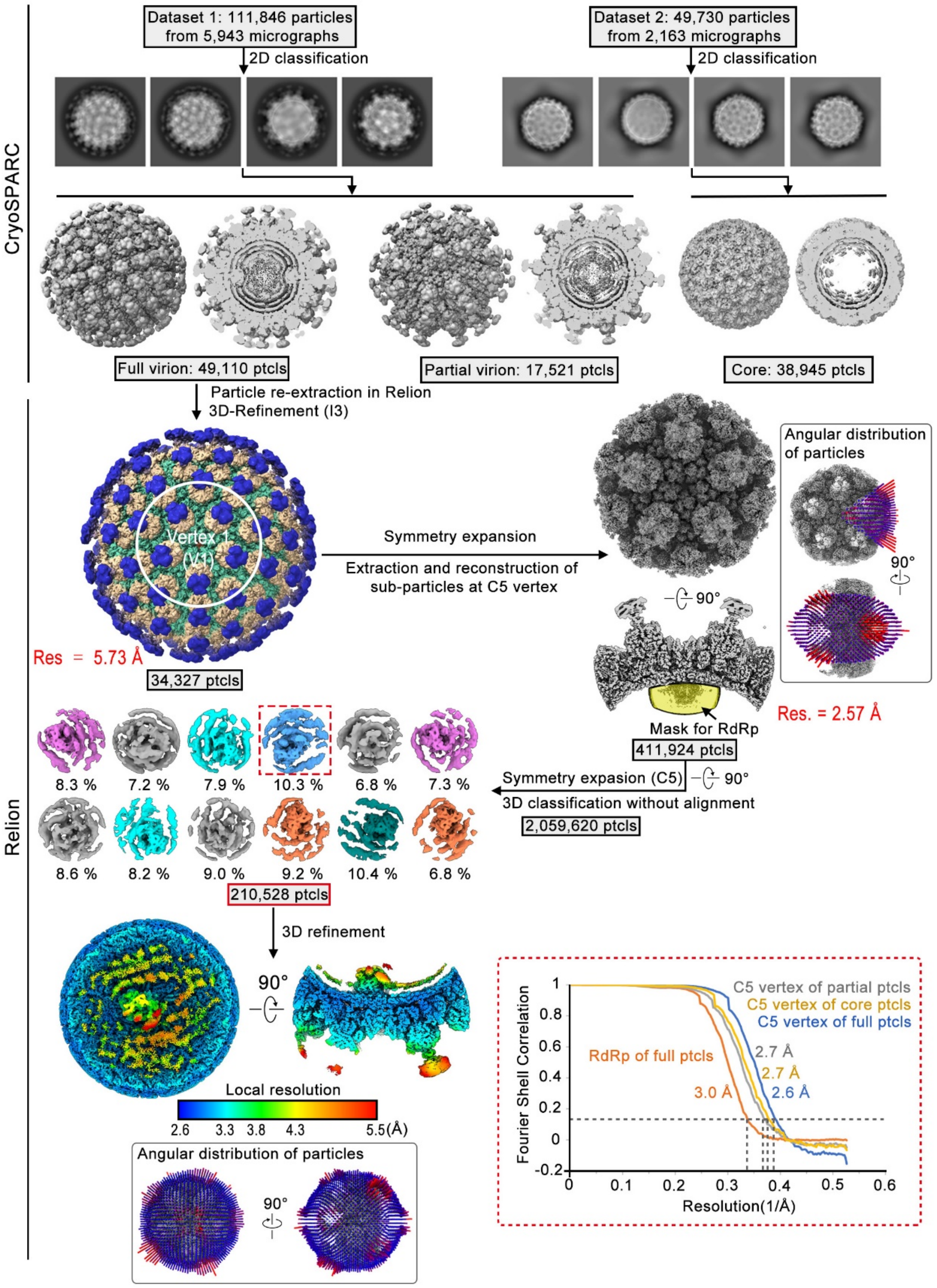

Supplementary Fig. 3 Strategies for local reconstructions at fivefold vertices and asymmetrical reconstruction of RdRp (VP1). For simplicity, data processing steps using Relion are shown only for full virions, and FSC curves for four cryo-EM structures are shown in the red box (bottom right).

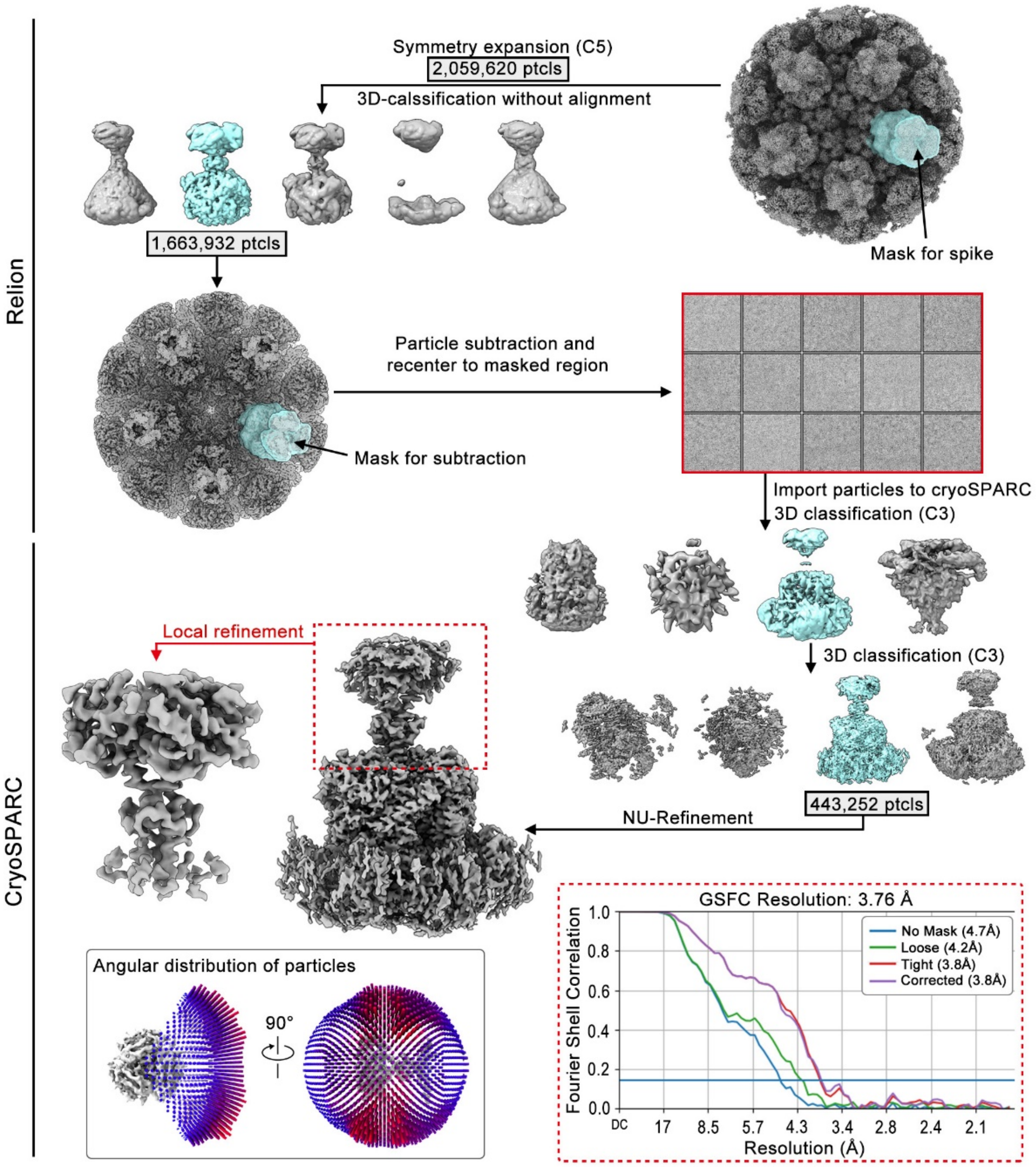

Supplementary Fig. 4 Strategy for local reconstruction of VP9.

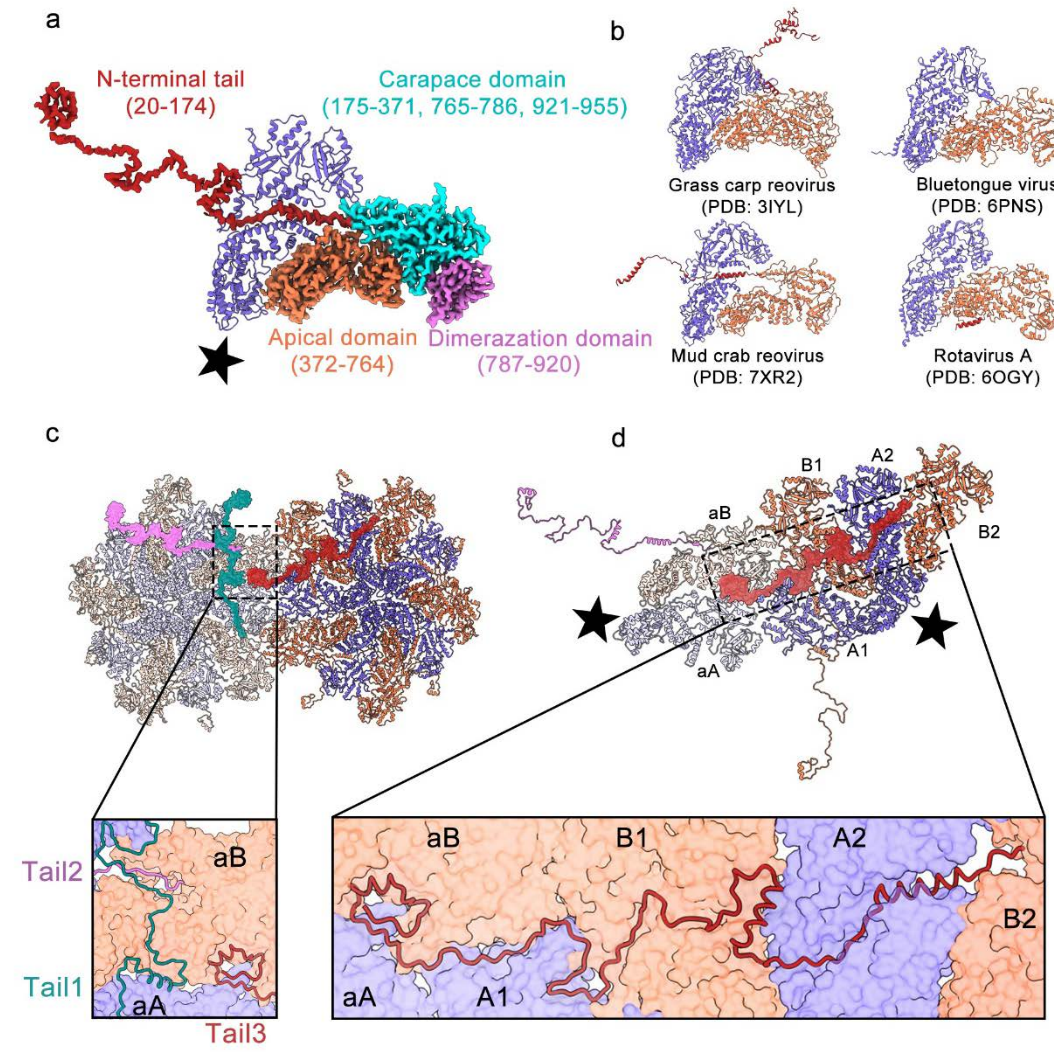

Supplementary Fig. 5 VP2A and VP2B viewed from inside of the virus.

a, Ribbon diagram of VP2A and VP2B heterodimer. The VP2A model is colored blue, while VP2B is color-coded by domain. The fivefold vertex is denoted by a five-pointed star.

b, Heterodimer of inner capsid protein from four other reoviruses. Compared with other VP2B-like molecules, the resolved *N*-terminal tail of BAV VP2B extends into neighboring VP2 molecules with different lengths and paths.

c, Three *N*-terminal tails (colored green, pink, and red) of VP2B highlighted in the context of two neighboring pentons. Tail 3 extends from one penton (the right one) into the neighboring penton (the left one). The inset shows that Tail 1 and Tail 3 interact with both VP2A (aA) and VP2B (aB).

d, VP2 subunits interacting with one *N*-terminal tail of VP2B. The *N*-terminal tail (red) of

one VP2B (labeled B2) interacts with three subunits (A1, A2 and B1) from one penton and two subunits (aA and aB) from the neighboring penton. Detailed information on the interactions between the tail and the five VP2 subunits (A1, A2, B1, aA, and aB) is given in Supplementary Table 2.

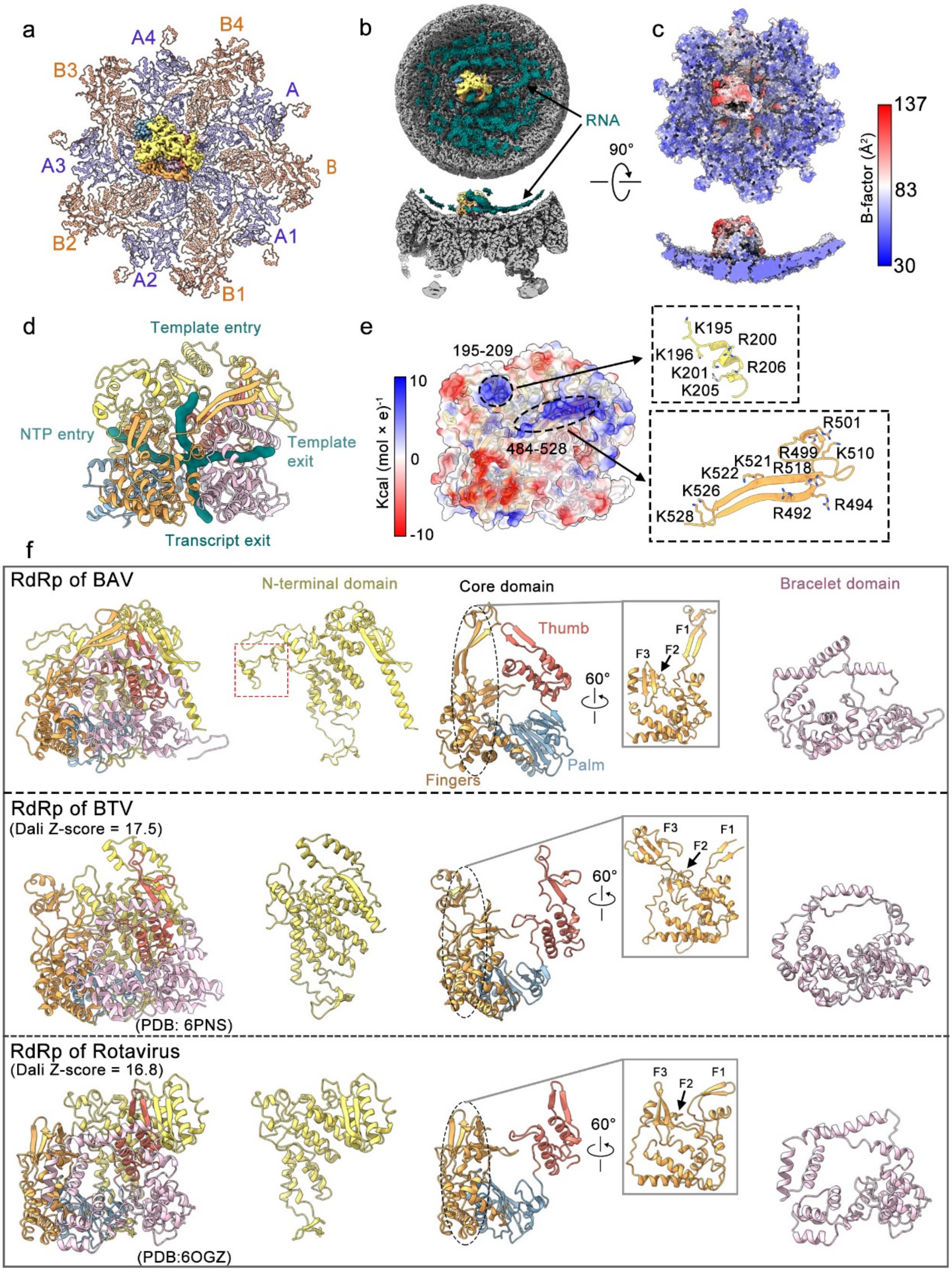

Supplementary Fig. 6 RdRp.

a, Atomic model of RdRp based on asymmetrical reconstruction at icosahedral vertices. VP2A and VP2B are numbered. Density for RdRp is colored by domain. The five VP2A molecules constituting the five-fold channel were resolved to the same number of residues (A–A4: 182–955, except one N-terminal extension shown in Fig. 2f). In the B

molecules, residues 404–428 were not resolved in the B, B1, B2, and B4 strands. Residues P404–T421 were resolved in the B3 chain due to the interaction with RdRp.

b, Orthogonal views of internal filamentous dsRNA density, colored in dark green.

c, Model of RdRp and nearby VP2 subunits. The map is color-coded by *B*-factors. Blue indicates a low *B*-factor, and red indicates a high *B*-factor.

d, Ribbon diagram of RdRp with the four channels [RNA template entry, nucleoside triphosphate (NTP) entry, template exit, and RNA transcript exit] indicated by dark green tubes.

e, Electrostatic potential diagram of RdRp. Two positively charged patches (marked by ellipses) near the RNA template entry site are in contact with dsRNA. No additional electron density is observed in the catalytic core of RdRp to place RNA.

f, Comparison of BAV RdRp with other sedoreovirus RdRps. BAV RdRp (top panel) consists of a *N*-terminal domain (residues 1–415), a core domain (residues 416–913), and a bracelet domain (residues 914–1219). There is a unique "fingernail" motif at the tip of the "fingers" subdomain in BTV RdRp (middle panel, the 'F3' finger in the core domain), which is mainly involved in interactions with the *N*-terminal domain. BAV has no such "fingernail" structure in the RdRp core region, instead showing an extended loop (residues 137–197, marked by a red box) in the *N*-terminal domain, which interacts with the fingers domain extensively. In addition, an extraordinarily long finger ('F1' finger, residues 484–528) over the bracelet domain is present in the core domain of BAV. A number of basic residues in the 'F1' finger are involved in binding with RNA near the template entry site (shown in **6e**).

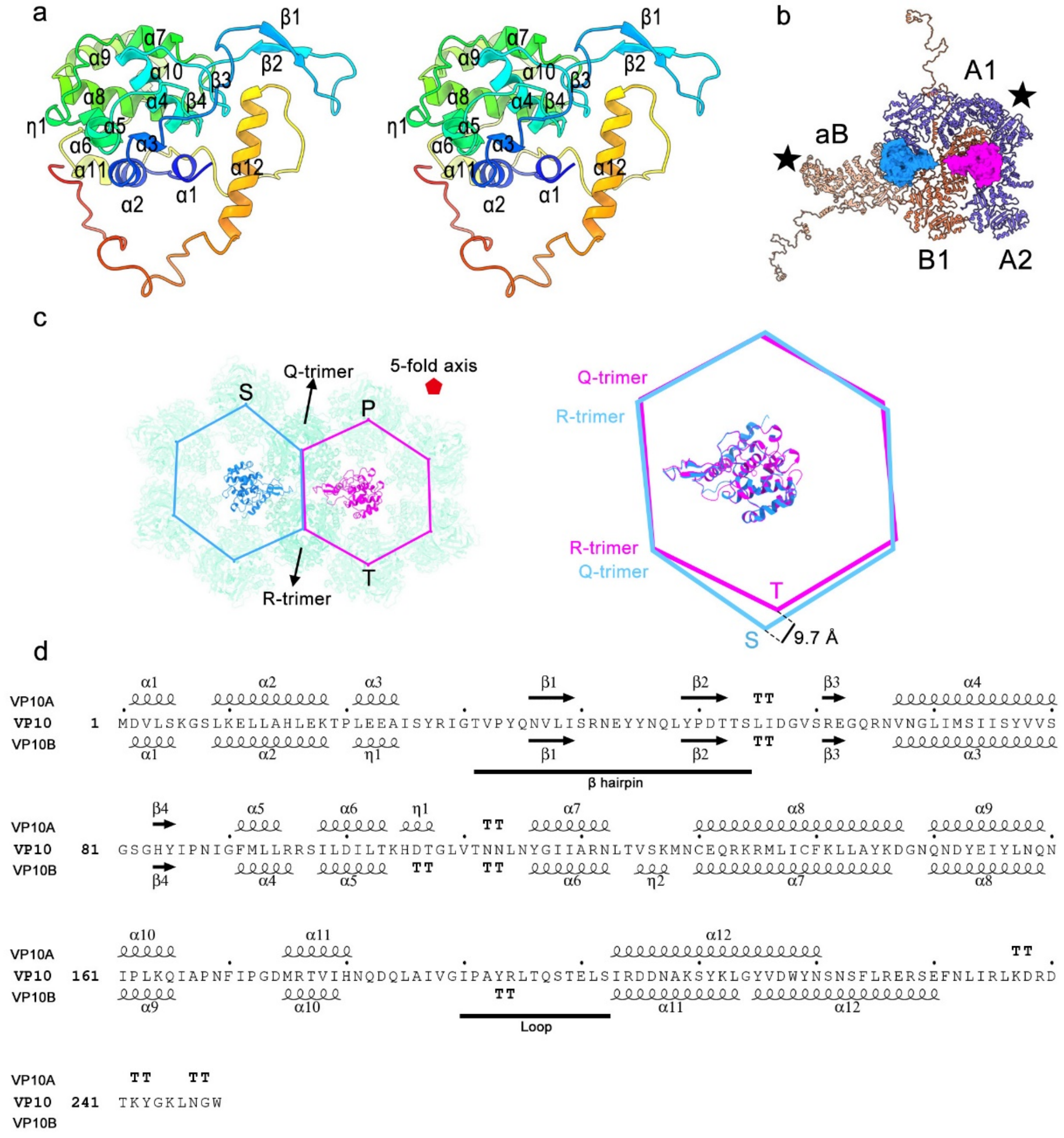

Supplementary Fig. 7 VP10.

a, Stereo view of ribbon diagram of the VP10A atomic model with secondary structural elements labeled. The model is rainbow-colored by residues from the *N*-terminus (blue) to the *C*-terminus (red).

b, Top view (from outside of the virus) of VP10A (magenta) and VP10B (blue) in the context of the inner capsid shell made up of VP2 molecules. Detailed information on the interactions between VP10 and VP2 (A1, A2, B1, and aB) is given in Supplementary Table 3.

c, Structural comparison of the type II and type III channels. VP10A (magenta) and VP10B (blue) are superimposed. The hexagons formed by six surrounding VP8 trimers are shown as cartoon lines connecting the centers-of-mass of the neighboring VP8 trimers.

d, Sequence of VP10. Secondary structure elements assigned by DSSP are indicated on top based on the VP10A structure, and at the bottom based on the VP10B structure.

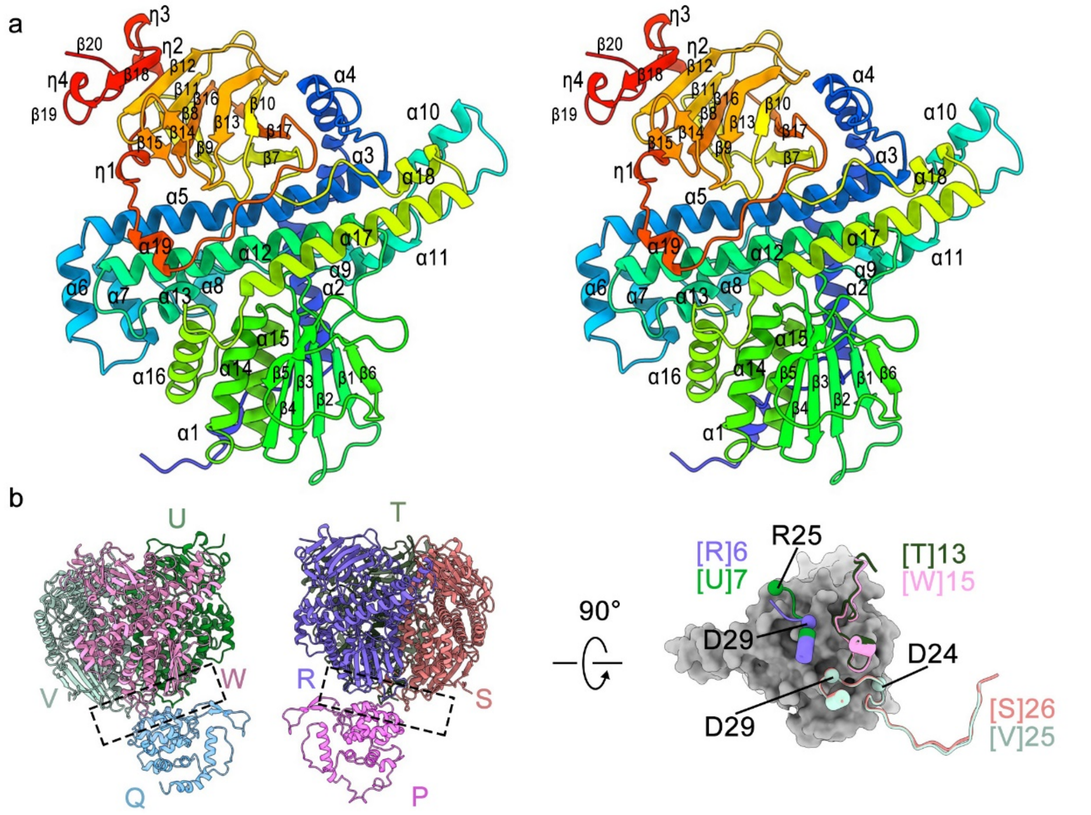

Supplementary Fig. 8 VP4.

a, Stereo view of ribbon diagram of the VP4 atomic model with secondary structural elements labeled. The model is rainbow-colored by residues from the *N*-terminus (blue) to the *C*-terminus (red).

b, VP4 (R, S, T, U, V, and W) and VP10 (P and Q) molecules in an ASU. (Right) *N*-terminal tails of VP4 were traced to different lengths. Negatively charged residues distributed on these tails are labeled. Detailed information on the interactions between VP10 and VP4 is given in Supplementary Table 5.

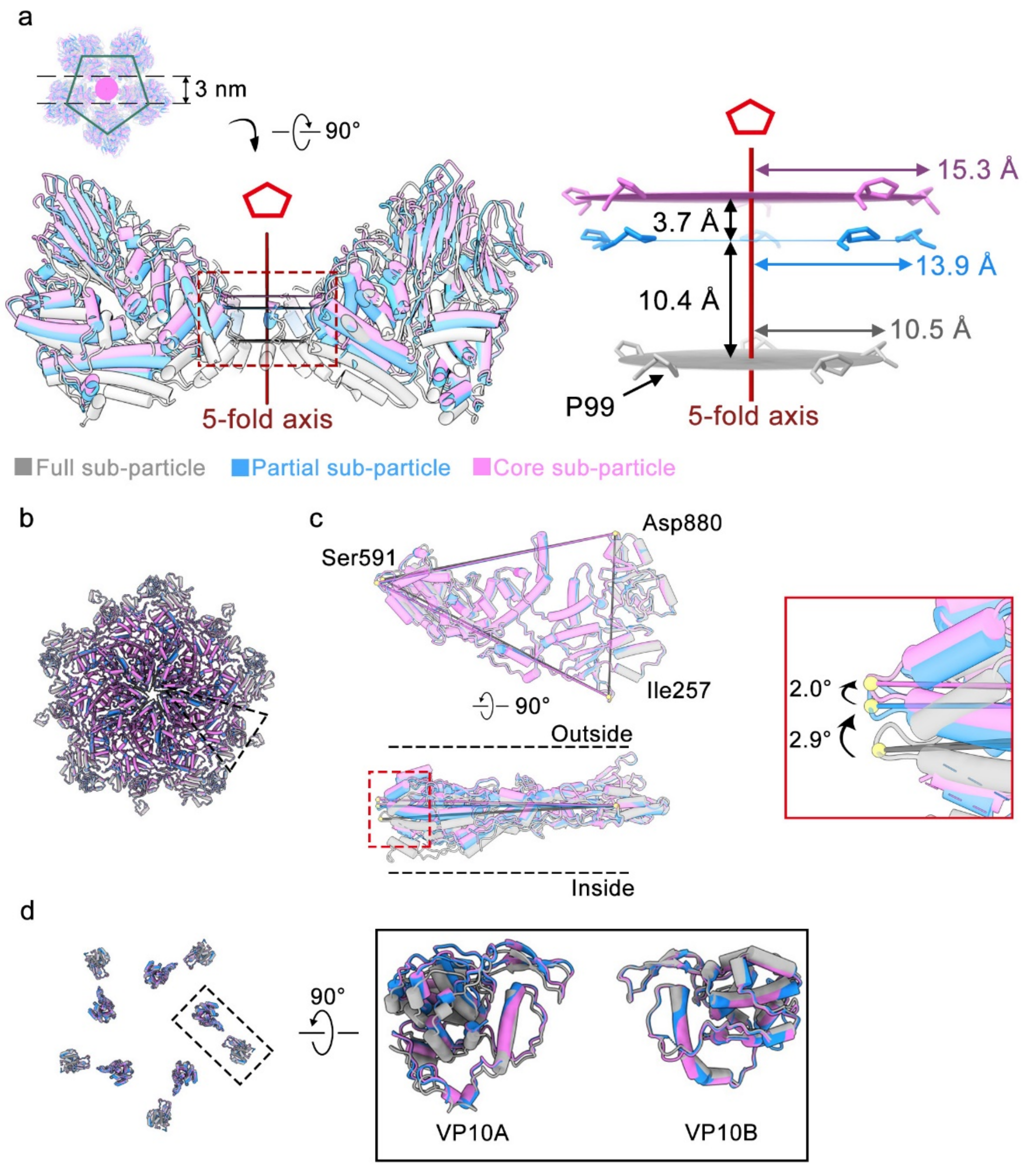

Supplementary Fig. 9 Structures of BAV particles in three states.

a, Side view of structural variations in the type I channel defined by five VP8 trimers near the fivefold axis. For simplicity, only the central slab of the channel (corresponding to a thickness of 3 nm) is shown for full (gray), partial (blue), and core (pink) particles (left panel). To compare the pore size of the channel, we measured the diameter of the circle defined by the Cβ atom of residue Pro99 from the five adjacent VP8 protomers (right panel). The measurement established that the opening of the channel in the partial particles adopts a size between that in the full and core structures.

b, Top view of structural variations in VP2 from three types of virions near the fivefold

axis.

c, (Left) Orthogonal views of the triangle region (boxed region in **a**) defined by the Cα atoms of residues S591, I257, and D880. (Right) Zoomed-in view of the red box. S591 of VP2 near the C5-fold axis in partial BAV particles rotates 2.9° outward relative to VP2 in the full BAV, and it rotates 2.0° further outward in the core conformation.

d, Top view of structural variations in VP10. VP10A moves more than VP10B does.

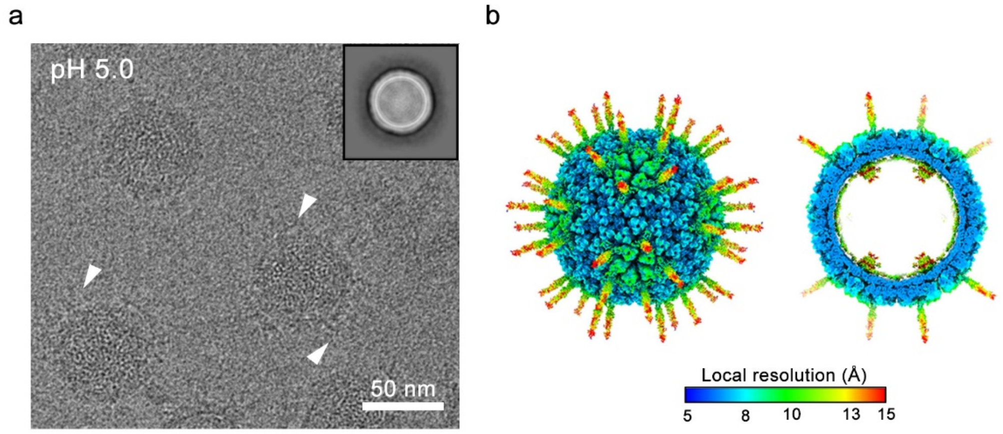

Supplementary Fig. 10 Cryo-EM structure of acidified BAV particles.

a, A representative cryo-EM micrograph of acidified BAV particles on ultrathin carbon film. Only one 2D class (shown in the inset) was identified based on ~1,400 particles. Projections on viral particles are indicated by white arrowheads.

b, Local resolution estimation of cryo-EM reconstruction of acidified BAV particles.

Supplementary Table 1. Cryo-EM data collection, refinement, and validation statistics.

**Refined models** [a]	C5 Vertex of full particles (EMD-36870) (PDB-8K42)	C5 Vertex of partial particles (EMD-36880) (PDB-8K49)	C5 Vertex of core particles (EMD-36881) (PDB-8K4A)	C1 Vertex of full particles showing VP1 (EMD-36871) (PDB-8K43)	Local reconstruction of VP9 (EMD-36872) (PDB-8K44)
**Data collection and processing**					
Magnification	50,000 ×	50,000 ×	50,000 ×	50,000 ×	50,000 ×
Voltage (kV)	300	300	300	300	300
Election exposure ($e^-/Å^2$)	40	40	40	40	40
Defocus range (μm)	-0.5 to -2.5	-0.5 to -2.5	-0.5 to -2.5	-0.5 to -2.5	-0.5 to -2.5
Pixel size (Å)	0.95	0.95	0.95	0.95	0.95
Symmetry imposed	C5	C5	C5	C1	C3
Final particle images (no.)	410,924	158,923	508,374	210,465	443,252
Map resolution (Å)	2.6	2.7	2.7	3.0	3.8
FSC threshold	0.143	0.143	0.143	0.143	0.143
**Model refinement**					
Model composition					
Chains	29	23	17	12	3
Non-hydrogen atoms	76,359	61,697	46,900	77,462	6,360
Protein residues	9,797	7,919	6,027	9,682	840
B factors ($Å^2$) (mean)					
Protein	56.24	63.47	57.52	66.00	118.28
R.m.s. deviations					
Bond lengths (Å)	0.003(0)	0.004(0)	0.003(0)	0.003(0)	0.003(0)
Bond angles (°)	0.476(0)	0.489(0)	0.536(0)	0.502(0)	0.531(0)
Validation					
MolProbity score	1.31	1.45	1.36	1.40	1.68
Clashscore	5.66	7.25	6.57	7.18	14.98
Poor rotamers (%)	0.42	1.15	0.27	0.38	0.41
Ramachandran plot					
Favored (%)	98.57	98.08	98.18	98.11	98.20
Allowed (%)	1.43	1.98	1.82	1.89	1.80
Disallowed (%)	0.00	0.00	0.00	0.00	0.00

[a] For icosahedral particle reconstructions, the map resolution was 5.7 Å (full particles, EMD-37378), 5.7 Å (partial particles, EMD-37379), or 4.8 Å (core particles, EMD-37380), respectively. Local reconstructions of fivefold vertices were used for model building, and the resulting atomic models were directly docked into the corresponding icosahedral reconstructions with PDB ID: 8W9P, 8W9Q, and 8W9R.

Supplementary Table 2. Interactions between an *N*-terminal tail of VP2B and nearby VP2 subunits.

Tail(B2)–VP2(A2)	Hydrogen bonds		
1	B2:ASN117[ND2]	3.07	A2:SER941[O]
2	B2:TYR125[OH]	3.36	A2:GLU362[OE1]
3	B2:TYR125[OH]	3.55	A2:GLU362[OE2]
4	B2:THR138[OG1]	3.7	A2:THR953[OG1]
5	B2:THR140[OG1]	3.11	A2:VAL951[O]
6	B2:PHE141[N]	3.07	A2:VAL951[O]
7	B2:GLN146[NE2]	2.79	A2:ASN373[O]
8	B2:LYS156[NZ]	3.25	A2:ALA725[O]
9	B2:TYR158[OH]	3.59	A2:ASN773[OD1]
10	B2:SER165[OG]	3.62	A2:SER220[OG]
11	B2:ASN167[ND2]	3.73	A2:ASN715[OD1]
12	B2:GLN118[OE1]	3.47	A2:TYR944[N]
13	B2:TYR125[OH]	3.18	A2:ARG356[NH2]
14	B2:GLN128[OE1]	3.01	A2:LYS359[NZ]
15	B2:VAL136[O]	2.31	A2:ARG354[NH2]
16	B2:LYS139[O]	2.86	A2:THR953[N]
17	B2:LYS139[O]	2.77	A2:THR953[OG1]
18	B2:GLU144[O]	3.72	A2:ARG370[NH2]
19	B2:GLU144[O]	3.73	A2:TYR341[OH]
20	B2:GLU144[OE1]	2.6	A2:TYR341[OH]
21	B2:GLN146[OE1]	2.62	A2:ARG549[NH1]
22	B2:SER165[OG]	3.84	A2:SER220[N]
	**Salt bridges**		
1	B2:GLU134[OE1]	3.63	A2:ARG357[NE]

Tail(B2)–VP2(B1)	Hydrogen bonds		
1	B2:ARG85[NH1]	2.87	B1:SER955[O]
2	B2:ILE86[N]	3.51	B1:VAL168[O]
3	B2:VAL89[N]	3.15	B1:SER171[O]
4	B2:LYS90[NZ]	3.15	B1:THR173[OG1]
5	B2:LEU91[N]	3.02	B1:THR173[O]
6	B2:ASP92[N]	3.18	B1:GLU676[OE2]
7	B2:GLU94[N]	3	B1:ASP679[OD2]
8	B2:GLN96[NE2]	2.81	B1:THR180[O]
9	B2:ASP99[N]	2.65	B1:GLU680[OE2]
10	B2:ILE132[N]	3.56	B1:SER390[O]
11	B2:THR72[O]	3.37	B1:ARG356[NH1]
12	B2:GLU73[OE2]	3.5	B1:ARG356[NH1]
13	B2:ILE86[O]	2.61	B1:SER170[OG]
14	B2:VAL89[O]	2.85	B1:THR173[N]
15	B2:LEU93[O]	3.55	B1:LYS177[NZ]
16	B2:GLU94[O]	2.86	B1:LYS177[NZ]
17	B2:GLU94[OE1]	3.02	B1:LYS177[N]
18	B2:GLU94[OE1]	2.75	B1:ARG178[N]
19	B2:GLU94[OE2]	2.55	B1:ARG549[NE]
20	B2:LYS95[O]	3.02	B1:THR683[OG1]
21	B2:GLN96[OE1]	3.76	B1:THR180[N]
22	B2:GLN96[OE1]	3.62	B1:GLY179[N]
23	B2:LYS102[O]	2.76	B1:LYS395[NZ]
24	B2:LYS102[O]	3.74	B1:TYR677[OH]
25	B2:PRO111[O]	3.39	B1:TYR749[OH]
26	B2:LEU112[O]	3.84	B1:ASN714[ND2]
27	B2:ILE114[O]	2.96	B1:ASN714[ND2]
28	B2:ASN117[OD1]	3.08	B1:LYS746[N]
29	B2:LEU124[O]	3.77	B1:TYR391[OH]
30	B2:THR127[O]	3.66	B1:TYR391[OH]
31	B2:SER130[O]	2.3	B1:LYS394[NZ]
32	B2:GLU134[O]	2.82	B1:ASN644[N]
33	B2:GLU134[OE1]	3.75	B1:GLU642[N]
	**Salt bridges**		
1	B2:ARG85[NH1]	2.87	B1:SER955[O]
2	B2:ARG85[NH1]	3.62	B1:SER955[OXT]
3	B2:ARG85[NH2]	3.58	B1:SER955[O]
4	B2:LYS90[NZ]	3.55	B1:GLU175[OE2]
5	B2:LYS95[NZ]	3.22	B1:GLU680[OE2]
6	B2:GLU73[OE2]	3.5	B1:ARG356[NH1]
7	B2:GLU94[OE2]	2.55	B1:ARG549[NE]
8	B2:GLU94[OE2]	3.76	B1:ARG549[NH2]

Tail(B2)–VP2(A1)	Hydrogen bonds		
1	B2:SER62[N]	3	A1:ASN909[O]
2	B2:ASP66[N]	3.41	A1:LEU876[O]
3	B2:VAL68[N]	3.26	A1:VAL878[O]
4	B2:THR72[N]	3.41	A1:ASP880[OD2]
5	B2:THR72[OG1]	2.85	A1:THR881[OG1]
6	B2:ALA80[N]	3.21	A1:ILE885[O]
7	B2:GLY84[N]	3.26	A1:THR217[O]
8	B2:GLY58[O]	3.4	A1:ASN909[ND2]
9	B2:LEU60[O]	3.66	A1:ARG870[NH2]
10	B2:SER62[O]	3.55	A1:SER911[OG]
11	B2:SER62[OG]	2.35	A1:ARG870[NH1]
12	B2:LEU64[O]	3.35	A1:ILE875[N]
13	B2:LEU64[O]	3.45	A1:LEU876[N]
14	B2:ASP66[O]	3.06	A1:VAL878[N]
15	B2:THR72[O]	2.92	A1:THR881[OG1]
16	B2:THR72[OG1]	3.64	A1:THR881[N]
17	B2:GLU73[O]	3.46	A1:THR881[OG1]
18	B2:ALA80[O]	3.04	A1:ASN857[N]
19	B2:ALA80[O]	3.1	A1:ASN857[ND2]
20	B2:LYS82[O]	2.96	A1:THR217[N]
21	B2:LYS82[O]	3.57	A1:THR217[OG1]
22	B2:ASP83[OD1]	3.08	A1:THR217[OG1]
	**Salt bridges**		
1	B2:ASP66[OD1]	3.62	A1:LYS886[NZ]

Tail(B2)–VP2(B3)	Hydrogen bonds		
1	B2:VAL168[O]	3.14	B3:ILE86[N]
2	B2:SER171[O]	3.5	B3:VAL89[N]
3	B2:THR173[O]	2.97	B3:LEU91[N]
5	B2:LYS166[NZ]	2.39	B3:GLY84[O]
6	B2:SER170[OG]	2.71	B3:ILE86[O]
7	B2:THR173[N]	3.34	B3:VAL89[O]

Tail(B2)–VP2(aB)	Hydrogen bonds		
1	B2:ALA28[O]	2.89	aB:ASN857[ND2]
2	B2:ALA28[O]	3.34	aB:ASN857[N]
3	B2:VAL29[O]	3.53	aB:THR217[N]
4	B2:VAL29[O]	2.95	aB:THR217[OG1]
5	B2:THR41[O]	2.86	aB:ASN715[ND2]
6	B2:TYR42[O]	2.77	aB:HIS712[ND1]
7	B2:SER43[O]	3.1	aB:THR705[N]
8	B2:SER43[O]	3.48	aB:SER706[N]
9	B2:ASN44[OD1]	3.7	aB:SER706[N]
10	B2:ASN44[OD1]	2.84	aB:SER706[OG]
11	B2:GLU53[O]	3.82	aB:ASP880[N]
12	B2:TYR56[O]	3.11	aB:VAL878[N]
13	B2:ASP57[OD1]	2.42	aB:LYS886[NZ]
14	B2:ALA65[O]	3.33	aB:ASN909[ND2]
15	B2:ALA28[N]	3.11	aB:ILE885[O]
16	B2:GLN30[NE2]	3.46	aB:GLU216[OE2]
17	B2:TYR42[OH]	2.44	aB:ASN223[OD1]
18	B2:LYS46[N]	3.21	aB:SER706[OG]
19	B2:LYS51[NZ]	3.36	aB:ASP884[OD2]
20	B2:GLU53[N]	3.68	aB:ASP880[OD1]
21	B2:GLU53[N]	3.7	aB:ASP880[OD2]
22	B2:ILE55[N]	2.94	aB:VAL878[O]
23	B2:TYR56[N]	3.15	aB:VAL878[O]
	**Salt bridges**		
1	B2:ASP57[OD1]	2.42	aB:LYS886[NZ]
2	B2:LYS51[NZ]	3.36	aB:ASP884[OD2]

Tail(B2)–VP2(aA)	Hydrogen bonds		
1	B2:ASN44[OD1]	3.11	aA:THR252[OG1]
2	B2:LYS46[O]	2.69	aA:THR252[N]
3	B2:LYS46[O]	2.5	aA:THR252[OG1]
4	B2:GLU53[OE1]	3.82	aA:ARG270[NH1]
5	B2:GLN30[NE2]	3.33	aA:THR254[O]
6	B2:GLN47[NE2]	2.58	aA:ASP253[O]
7	B2:ARG48[N]	3.26	aA:THR252[O]
	**Salt bridges**		
1	B2:GLU53[OE1]	3.82	aA:ARG270[NH1]
2	B2:GLU53[OE2]	3.93	aA:ARG270[NH1]

Supplementary Table 3. Interactions between VP10A/VP10B and VP2.

VP10A(P)–VP2(B1) [a]	Hydrogen bonds		
1	P:SER221[O]	2.71	B1:ARG460[NH2]
2	P:ASN222[O]	3.44	B1:SER490[OG]
3	P:ASN222[O]	3.33	B1:ASP491[N]
4	P:PHE224[O]	2.54	B1:ARG460[NH2]
5	P:GLU230[OE1]	3.35	B1:SER490[OG]
6	P:GLU230[OE1]	3.3	B1:ARG460[NH1]
7	P:LEU233[O]	3.48	B1:ARG495[NH2]
8	P:ASN222[ND2]	3.06	B1:ALA486[O]
9	P:ASN222[ND2]	3.48	B1:ILE489[O]
10	P:ARG226[NH1]	3.11	B1:GLY641[O]
11	P:ARG226[NH2]	2.62	B1:GLN458[O]
12	P:ARG235[NH1]	3.77	B1:ASN514[O]
13	P:ARG235[NH1]	3.47	B1:ASP516[O]
	**Salt bridges**		
1	P:GLU230[OE1]	3.3	B1:ARG460[NH1]
**VP10A(P)–VP2(A2)**	**Hydrogen bonds**		
1	P:SER229[OG]	2.37	A2:ARG357[NH2]
2	P:ASP240[O]	2.88	A2:THR346[OG1]
3	P:ASP240[OD2]	3.65	A2:ARG343[NH1]
4	P:ARG228[NH2]	2.92	A2:GLU360[OE2]
5	P:ASN232[ND2]	3.32	A2:TYR353[O]
6	P:ARG239[NH1]	3.07	A2:THR346[O]
7	P:ARG239[NH1]	3.29	A2:VAL349[O]
8	P:LYS242[N]	3.81	A2:ARG343[O]
9	P:TYR243[OH]	3.48	A2:GLU344[OE2]
	**Salt bridges**		
1	P:ASP240[OD2]	3.65	A2:ARG343[NH1]
2	P:ARG228[NH2]	3.55	A2:GLU360[OE1]
3	P:ARG228[NH2]	2.92	A2:GLU360[OE2]

VP10B(Q)–VP2(A1)	Hydrogen bonds		
1	Q:LYS134[NZ]	3.65	A1:ASN825[OD1]
2	Q:ASN169[ND2]	3.88	A1:MET823[O]
3	Q:GLN182[NE2]	2.97	A1:GLU847[O]
**VP10B(Q)–VP2(B1)**	**Hydrogen bonds**		
1	Q:ARG194[O]	3.82	B1:LYS351[NZ]
2	Q:ASP217[OD1]	3.24	B1:ARG343[NE]
3	Q:ARG228[NH2]	3.76	B1:ASP307[OD1]
	**Salt bridges**		
1	Q:ASP217[OD1]	3.24	B1:ARG343[NE]
2	Q:ASP217[OD1]	3.86	B1:ARG343[NH2]
3	Q:ARG228[NH2]	3.76	B1:ASP307[OD1]
**VP10B(Q)–VP2(aB)**	**Hydrogen bonds**		
1	Q:GLU227[OE1]	3.31	aB:LYS202[NZ]
2	Q:ARG228[O]	3.41	aB:ARG285[NH1]
3	Q:ARG228[NE]	3.16	aB:TYR283[O]
4	Q:ARG228[NH2]	2.65	aB:ASP284[O]
	**salt bridges**		
1	Q:GLU227[OE1]	3.31	aB:LYS202[NZ]
2	Q:GLU227[OE2]	3.67	aB:LYS202[NZ]

[a] Chain numbers are indicated in Supplementary Fig. 7b.

Supplementary Table 4. Interactions between VP8 (Q- or R-trimer) and VP10.

VP10A(P)–VP8(Q3) [a]	Hydrogen bonds		
1	P:GLY30[O]	3	Q3:ASN19[ND2]
2	P:THR31[OG1]	3.61	Q3:ALA96[N]
3	P:LEU38[O]	3.14	Q3:ARG87[NH2]
4	P:SER40[O]	3.66	Q3:ASN80[ND2]
5	P:SER40[O]	3.52	Q3:ASN84[ND2]
6	P:SER210[OG]	3.1	Q3:LYS77[NZ]
7	P:GLY30[N]	3.22	Q3:ASN19[OD1]
8	P:ARG28[NH1]	3.02	Q3:ASP22[OD2]
9	P:ARG41[NH1]	3.18	Q3:ASN80[OD1]
10	P:SER40[N]	3.28	Q3:ASN84[OD1]
11	P:ARG61[NH1]	2.66	Q3:ALA96[O]
	**Salt bridges**		
1	P:ARG28[NH1]	3.02	Q3:ASP22[OD2]
**VP10A(P)–VP8(R2)**	**Hydrogen bonds**		
1	P:ASN46[OD1]	3.07	R2:ARG300[NH1]
2	P:ASN42[OD1]	3.88	R2:ARG300[NH1]
3	P:ASN42[O]	2.79	R2:ARG300[NH2]
4	P:ARG194[NH2]	3.63	R2:ASP47[O]
5	P:ARG194[NH1]	3.74	R2:ASP47[O]
6	P:ARG194[NH2]	2.92	R2:GLU48[O]
**VP10A(P)–VP8(R3)**	**Hydrogen bonds**		
1	P:SER126[O]	3.75	R3:ASN19[ND2]
2	P:MET128[O]	3.52	R3:ASN19[ND2]
3	P:GLU200[OE2]	3.08	R3:GLU23[N]
4	P:GLU200[OE1]	3.01	R3:ARG26[NH1]
5	P:ARG194[O]	2.72	R3:GLN27[NE2]
6	P:GLU131[OE1]	3.55	R3:GLN100[N]
7	P:GLU131[OE2]	3.44	R3:GLN100[N]
8	P:TYR45[OH]	3.7	R3:TYR117[OH]
9	P:PRO50[O]	2.6	R3:ASN275[ND2]
10	P:LYS212[NZ]	3.68	R3:ASP22[OD2]
11	P:THR196[N]	3.17	R3:GLU23[OE1]
12	P:THR196[OG1]	2.71	R3:GLU23[OE2]
13	P:ARG194[N]	3.31	R3:GLN27[OE1]
14	P:CYS130[SG]	3.82	R3:ILE97[O]
15	P:ARG194[NH2]	3.46	R3:THR111[OG1]
16	P:TYR49[OH]	3.88	R3:ASN275[O]
	**Salt bridges**		
1	P:GLU200[OE1]	3.01	R3:ARG26[NH1]
2	P:GLU200[OE2]	3.79	R3:ARG26[NH1]
3	P:LYS212[NZ]	3.68	R3:ASP22[OD2]
4	P:LYS209[NZ]	3.53	R3:GLU23[OE1]

VP10B(Q)–VP8(Q1)	Hydrogen bonds		
1	Q:GLU200[OE2]	3.5	Q1:GLU23[N]
2	Q:GLU200[OE1]	3.77	Q1:GLU23[N]
3	Q:GLU200[OE1]	2.69	Q1:ARG26[NH2]
4	Q:ARG194[O]	3.46	Q1:GLN27[NE2]
5	Q:GLU131[OE2]	3.04	Q1:GLN100[N]
6	Q:TYR45[OH]	3.39	Q1:TYR117[OH]
7	Q:THR196[N]	3.15	Q1:GLU23[OE1]
8	Q:THR196[OG1]	3.21	Q1:GLU23[OE1]
9	Q:ARG133[NH1]	3.85	Q1:PRO95[O]
10	Q:ARG194[NH1]	2.93	Q1:ASP104[O]
11	Q:ARG194[NH1]	2.73	Q1:SER108[OG]
12	Q:TYR49[OH]	3.76	Q1:ASN275[O]
	**Salt bridges**		
1	Q:GLU200[OE1]	2.69	Q1:ARG26[NH2]
2	Q:LYS209[NZ]	3.22	Q1:GLU23[OE1]
**VP10B(Q)–VP8(Q3)**	**Hydrogen bonds**		
1	Q:ASN42[OD1]	3.61	Q3:ARG300[NH1]
2	Q:ASN46[OD1]	3.07	Q3:ARG300[NH1]
3	Q:ASN42[O]	3.18	Q3:ARG300[NH1]
4	Q:ASN42[O]	2.94	Q3:ARG300[NH2]
5	Q:GLU43[O]	3.37	Q3:ARG300[NH2]
**VP10B(Q)–VP8(R2)**	**Hydrogen bonds**		
1	Q:GLY30[O]	3.72	R2:ASN19[ND2]
2	Q:THR31[OG1]	3.86	R2:ALA96[N]
3	Q:LEU38[O]	3.05	R2:ARG87[NH2]
4	Q:SER40[O]	3.55	R2:ASN84[ND2]
5	Q:SER40[O]	3.42	R2:ASN80[ND2]
6	Q:SER40[OG]	3.4	R2:THR83[OG1]
7	Q:SER210[OG]	2.9	R2:LYS77[NZ]
8	Q:GLN35[NE2]	3.01	R2:ASN93[O]
9	Q:SER40[N]	3.33	R2:ASN84[OD1]
10	Q:ARG41[NH1]	2.93	R2:ASN80[OD1]
11	Q:ARG61[NH1]	2.95	R2:ALA96[O]
12	Q:ARG61[NH2]	3.18	R2:ALA96[O]
	**Salt bridges**		
1	Q:ARG28[NH1]	3.83	R2:ASP22[OD1]
2	Q:ARG28[NH1]	3.61	R2:ASP22[OD2]

[a] Chain numbers are indicated in Fig. 3c.

Supplementary Table 5. Interactions between VP10A/VP10B and VP4 trimer.

VP10A(P)–VP4(R) [a]	Hydrogen bonds		
1	P:LEU114[O]	3.49	R:TYR286[OH]
2	P:GLN64[NE2]	3.27	R:ALA28[O]
3	P:ASN66[ND2]	2.72	R:ASP29[O]
4	P:TYR116[N]	3.53	R:TYR286[OH]
5	P:ARG121[NH1]	2.91	R:SER370[O]
6	P:ARG121[NH1]	3.34	R:LEU372[O]
**VP10A(P)–VP4(S)**	**Hydrogen bonds**		
1	P:ASN113[O]	3.24	S:ASP29[N]
2	P:ASN112[ND2]	3.77	S:THR26[OG1]
3	P:ASN113[ND2]	3.25	S:ASP29[OD2]
4	P:ASN149[ND2]	2.52	S:ASP24[OD2]
**VP10A(P)–VP4(T)**	**Hydrogen bonds**		
1	P:VAL67[N]	3.09	T:THR26[OG1]
**VP10B(Q)–VP4(U)**	**Hydrogen bonds**		
1	Q:GLU 62[OE2]	2.69	U:ARG 25[NH2]
2	Q:LEU 114[O]	3.50	U:TYR 286[OH]
3	Q:GLN 64[NE2]	3.26	U:ALA 28[O]
4	Q:TYR 116[N]	3.46	U:TYR 286[OH]
5	Q:ARG 121[NH1]	3.46	U:ASP 310[OD2]
6	Q:ARG 121[NH2]	3.37	U:ASP 310[OD2]
7	Q:ARG 121[NH1]	2.95	U:SER 370[O]
8	Q:ARG 121[NH2]	3.43	U:GLN 374[OE1]
	**Salt bridges**		
1	Q:GLU 62[OE1]	3.46	U:ARG 25[NH2]
2	Q:GLU 62[OE2]	2.69	U:ARG 25[NH2]
3	Q:ARG 121[NH1]	3.46	U:ASP 310[OD2]
4	Q:ARG 121[NH2]	3.37	U:ASP 310[OD2]
**VP10B(Q)–VP4(V)**	**Hydrogen bonds**		
1	Q:ASN 113[O]	3.25	V:ASP 29[N]
2	Q:ASN 113[ND2]	3.24	V:ASP 29[OD2]
3	Q:ASN 115[ND2]	2.70	V:ASP 29[O]
4	Q:ASN 115[ND2]	3.74	V:GLY 30[O]
5	Q:ASN 149[ND2]	3.44	V:ASP 24[OD2]
**VP10B(Q)–VP4(W)**	**Hydrogen bonds**		
1	Q:GLU 62[O]	2.75	W:TYR 286[OH]
2	Q:GLN 64[N]	3.30	W:TYR 286[OH]
3	Q:ARG 65[NH2]	3.28	W:ASN 23[O]
4	Q:VAL 67[N]	3.29	W:THR 26[OG1]

[a] Chain numbers are indicated in Supplementary Fig. 8b.
