# Supplementary material for: Cryo-EM structures of Banna virus in multiple states reveal stepwise detachment of viral spikes
Source: Nat Commun. 2024 Mar 13;15:2284. doi: 10.1038/s41467-024-46624-x (PMC10937716; doi:10.1038/s41467-024-46624-x)
Supplement: Supplementary file 3 — Reporting Summary [file 41467_2024_46624_MOESM3_ESM.pdf]

nature portfolio

Corresponding author(s): Sheng Cao

Last updated by author(s): Feb 20, 2024

Reporting Summary

Nature Portfolio wishes to improve the reproducibility of the work that we publish. This form provides structure for consistency and transparency in reporting. For further information on Nature Portfolio policies, see our Editorial Policies and the Editorial Policy Checklist.

Statistics

For all statistical analyses, confirm that the following items are present in the figure legend, table legend, main text, or Methods section.

n/a	Confirmed	
☐	☒	The exact sample size (*n*) for each experimental group/condition, given as a discrete number and unit of measurement
☒	☐	A statement on whether measurements were taken from distinct samples or whether the same sample was measured repeatedly
☒	☐	The statistical test(s) used AND whether they are one- or two-sided<br>*Only common tests should be described solely by name; describe more complex techniques in the Methods section.*
☒	☐	A description of all covariates tested
☒	☐	A description of any assumptions or corrections, such as tests of normality and adjustment for multiple comparisons
☐	☒	A full description of the statistical parameters including central tendency (e.g. means) or other basic estimates (e.g. regression coefficient) AND variation (e.g. standard deviation) or associated estimates of uncertainty (e.g. confidence intervals)
☒	☐	For null hypothesis testing, the test statistic (e.g. *F*, *t*, *r*) with confidence intervals, effect sizes, degrees of freedom and *P* value noted<br>*Give P values as exact values whenever suitable.*
☒	☐	For Bayesian analysis, information on the choice of priors and Markov chain Monte Carlo settings
☒	☐	For hierarchical and complex designs, identification of the appropriate level for tests and full reporting of outcomes
☒	☐	Estimates of effect sizes (e.g. Cohen's *d*, Pearson's *r*), indicating how they were calculated

Our web collection on statistics for biologists contains articles on many of the points above.

Software and code

Policy information about availability of computer code

Data collection	SerialEM 4.0
Data analysis	RELION 3.1, CryoSPARC v4, UCSF ChimeraX 1.6, PHENIX 1.19, Coot 0.9, MotionCorr2, AlphaFold2, EMAN2, DeepEMhancer, DSSP 3.0

For manuscripts utilizing custom algorithms or software that are central to the research but not yet described in published literature, software must be made available to editors and reviewers. We strongly encourage code deposition in a community repository (e.g. GitHub). See the Nature Portfolio guidelines for submitting code & software for further information.

Data

Policy information about availability of data

All manuscripts must include a data availability statement. This statement should provide the following information, where applicable:

- Accession codes, unique identifiers, or web links for publicly available datasets
- A description of any restrictions on data availability
- For clinical datasets or third party data, please ensure that the statement adheres to our policy

The full genome sequences of BAV YN15-126-01 were submitted to GenBank, and the accession numbers for segments 1 to 12 are OR004518 [https://www.ncbi.nlm.nih.gov/nuccore/OR004518], OR004519 [https://www.ncbi.nlm.nih.gov/nuccore/OR004519], OR004520 [https://www.ncbi.nlm.nih.gov/nuccore/OR004520], OR004521 [https://www.ncbi.nlm.nih.gov/nuccore/OR004521], OR004522 [https://www.ncbi.nlm.nih.gov/nuccore/OR004522], OR004523 [https://www.ncbi.nlm.nih.gov/nuccore/OR004523], OR004524 [https://www.ncbi.nlm.nih.gov/nuccore/OR004524], OR004525 [https://www.ncbi.nlm.nih.gov/nuccore/OR004525], OR004526 [https://www.ncbi.nlm.nih.gov/nuccore/OR004526], OR004527 [https://www.ncbi.nlm.nih.gov/nuccore/OR004527], OR004528 [https://

www.ncbi.nlm.nih.gov/nuccore/OR004528], OR004529 [https://www.ncbi.nlm.nih.gov/nuccore/OR004529]. Cryo-EM density maps have been deposited in the Electron Microscopy Data Bank under accession codes EMD-37378 [https://www.ebi.ac.uk/emdb/EMD-37378] (icosahedral full particles), EMD-37379 [https://www.ebi.ac.uk/emdb/EMD-37379] (icosahedral partial particles), EMD-37380 [https://www.ebi.ac.uk/emdb/EMD-37380] (icosahedral core particles), EMD-36870 [https://www.ebi.ac.uk/emdb/EMD-36870] (C5 vertex of full particles), EMD-36880 [https://www.ebi.ac.uk/emdb/EMD-36880] (C5 vertex of partial particles), EMD-36881 [https://www.ebi.ac.uk/emdb/EMD-36881] (C5 vertex of core particles), EMD-36871 [https://www.ebi.ac.uk/emdb/EMD-36871] (C1 vertex of full particle showing VP1), and EMD-36872 [https://www.ebi.ac.uk/emdb/EMD-36872] (local reconstruction of VP9). The corresponding atomic models have been deposited in the Protein Data Bank with accession codes 8W9P [http://doi.org/10.2210/pdb8W9P/pdb], 8W9Q [http://doi.org/10.2210/pdb8W9Q/pdb], 8W9R [http://doi.org/10.2210/pdb8W9R/pdb], 8K42 [http://doi.org/10.2210/pdb8K42/pdb], 8K49 [http://doi.org/10.2210/pdb8K49/pdb], 8K4A [http://doi.org/10.2210/pdb8K4A/pdb], 8K43 [http://doi.org/10.2210/pdb8K43/pdb], and 8K44 [http://doi.org/10.2210/pdb8K44/pdb], respectively. Previous reported atomic model of BTV VP5 can be accessed under accession code 3J9E [http://doi.org/10.2210/pdb3J9E/pdb].

Research involving human participants, their data, or biological material

Policy information about studies with human participants or human data. See also policy information about sex, gender (identity/presentation), and sexual orientation and race, ethnicity and racism.

Reporting on sex and gender	N/A.
Reporting on race, ethnicity, or other socially relevant groupings	N/A.
Population characteristics	N/A.
Recruitment	N/A.
Ethics oversight	N/A.

Note that full information on the approval of the study protocol must also be provided in the manuscript.

Field-specific reporting

Please select the one below that is the best fit for your research. If you are not sure, read the appropriate sections before making your selection.

☒ Life sciences ☐ Behavioural & social sciences ☐ Ecological, evolutionary & environmental sciences

For a reference copy of the document with all sections, see nature.com/documents/nr-reporting-summary-flat.pdf

Life sciences study design

All studies must disclose on these points even when the disclosure is negative.

Sample size	No statistical estimation was performed to predetermine sample sizes for our cryo-EM studies. Cryo-EM data collection was performed to obtain a sufficient number of particles for reliable classification and 3D reconstructions.
Data exclusions	Particle images with poor quality were excluded after 2D or 3D classification, based on the generally accepted principle in the cryo-EM field.
Replication	Results of biochemical experiments (e.g., SDS-PAGE and negative-stain TEM in Fig. 5) are representative of at least three independent experiments.
Randomization	Cryo-EM datasets were randomly split into two half sets for calculation of cross correlation coefficients. The randomization procedure was performed automatically in RELION or cryoSPARC.
Blinding	Blinding is not applicable to this study. No human subjects participated in this study.

Reporting for specific materials, systems and methods

We require information from authors about some types of materials, experimental systems and methods used in many studies. Here, indicate whether each material, system or method listed is relevant to your study. If you are not sure if a list item applies to your research, read the appropriate section before selecting a response.

Materials & experimental systems

n/a	Involved in the study
☒	☐ Antibodies
☐	☒ Eukaryotic cell lines
☒	☐ Palaeontology and archaeology
☒	☐ Animals and other organisms
☒	☐ Clinical data
☒	☐ Dual use research of concern
☒	☐ Plants

Methods

n/a	Involved in the study
☒	☐ ChIP-seq
☒	☐ Flow cytometry
☒	☐ MRI-based neuroimaging

Eukaryotic cell lines

Policy information about cell lines and Sex and Gender in Research

Cell line source(s)	LMH was kindly gifted from Prof. Meilin Jin (Huazhong Agricultural University), C6/36, BHK-21, PK15, Vero, A549, Huh-7 cells were from Cell bank in National Virus Resource Center of Wuhan Institute of Virology.
Authentication	Morphology check by microscope and growth curve analysis.
Mycoplasma contamination	All cell lines tested negative for Mycoplasma contamination.
Commonly misidentified lines (See ICLAC register)	No misidentified cell line has been used.

Plants

Seed stocks	*Report on the source of all seed stocks or other plant material used. If applicable, state the seed stock centre and catalogue number. If plant specimens were collected from the field, describe the collection location, date and sampling procedures.*
Novel plant genotypes	*Describe the methods by which all novel plant genotypes were produced. This includes those generated by transgenic approaches, gene editing, chemical/radiation-based mutagenesis and hybridization. For transgenic lines, describe the transformation method, the number of independent lines analyzed and the generation upon which experiments were performed. For gene-edited lines, describe the editor used, the endogenous sequence targeted for editing, the targeting guide RNA sequence (if applicable) and how the editor was applied.*
Authentication	*Describe any authentication procedures for each seed stock used or novel genotype generated. Describe any experiments used to assess the effect of a mutation and, where applicable, how potential secondary effects (e.g. second site T-DNA insertions, mosiacism, off-target gene editing) were examined.*
